# Age and cohort trends of the impact of socioeconomic status on dietary diversity among Chinese older adults from the perspective of urban–rural differences: A prospective cohort study based on CLHLS 2002–2018

**DOI:** 10.3389/fnut.2022.1020364

**Published:** 2022-10-20

**Authors:** Yan Yu, Na Cao, Anqi He, Junfeng Jiang

**Affiliations:** ^1^Wuhan Jinyintan Hospital, Tongji Medical College of Huazhong University of Science and Technology, Hubei Clinical Research Center for Infectious Diseases, Wuhan Research Center for Communicable Disease Diagnosis and Treatment, Chinese Academy of Medical Sciences, Joint Laboratory of Infectious Diseases and Health, Wuhan Institute of Virology and Wuhan Jinyintan Hospital, Chinese Academy of Sciences, Wuhan, China; ^2^School of Public Health, Wuhan University, Wuhan, China; ^3^BeiGene, Wuhan, China; ^4^School of Sociology, Central China Normal University, Wuhan, China

**Keywords:** dietary diversity score, socioeconomic status, age and cohort effects, older adults, rural and urban China

## Abstract

The association between socioeconomic status (SES) and dietary diversity score (DDS) has been widely discussed, but little is known about the age and cohort effects on DDS and how the SES effect on DDS varies with age and across successive cohorts among urban and rural older adults in China. Thus, this study aimed to examine the temporal change in DDS among Chinese older adults and SES heterogeneities in such change from the perspective of urban–rural differentiation. Data from the Chinese Longitudinal Healthy Longevity Survey (CLHLS) between 2002 and 2018 were used, and a total of 13,728 participants aged between 65 and 105 years were included in this study. A total of eight food groups were used to assess DDS, while education, family income, and perceived income status were used to assess SES. A linear mixed model was used to estimate the age and cohort effects on DDS and their urban–rural and SES disparities. The results show that higher SES, including more education, family income, and perceived income status, was associated with higher DDS (for urban older adults, β = 0.1645, *p* = 0.0003, β = 0.2638, *p* < 0.0001, β = 0.2917, *p* < 0.0001, respectively; for rural older adults, β = 0.0932, *p* = 0.0080, β = 0.4063, *p* < 0.0001, β = 0.2921, *p* < 0.0001, respectively). The DDS of older adults increased with age and across successive cohorts in both urban and rural China. Moreover, we found the three-way interaction effect of SES, age, and cohort was statistically significant in both urban and rural China. Thus, living in an urban area and having higher SES are associated with higher DDS, but these associations change with age and across successive cohorts. The dietary health of earlier cohorts and rural oldest-old in China deserves more attention.

## Introduction

Aging is one of the prominent features of the current international population, especially in developed countries and some developing countries. China experienced an increase in birth rate in the 1960s, accordingly it was theorized that the aging of the Chinese population will reach its peak in the 2020s (1). According to the Chinese Seventh National Census, the number of older adults aged 65 years or above in China had reached 191 million by the end of 2020, accounting for 13.5% of the total population (2). With the advent of aging society, the number of older adults in China has become increasingly large, which has greatly increased the burden of disease on individuals and society. Unhealthy diet and insufficient nutrition intake are important reasons for the poor health and diseases of older adults, which suggests that a healthy diet is important to reduce the burden of diseases and improve older adults’ health (3, 4).

Dietary diversity is an important measure of nutrient intake and dietary health (5). A diversified healthy eating pattern is strongly associated with better physical performance, reduced mortality and depression, and higher cognitive abilities and quality of life (6–15). Older adults’ dietary diversity is closely related to their socioeconomic status (SES). Previous studies have documented that more education and a better economic condition are associated with higher dietary diversity score (DDS) and healthier diet among older adults (16–19). However, some studies have also found that there is no significant association between SES and dietary diversity (6, 12, 20). In addition, there are significant differences in dietary habits and conditions between urban and rural older adults. More complete infrastructures and food supply systems in urban areas make the DDS of urban older adults significantly higher than that of their rural counterparts (17, 19, 21).

Previous studies have focused on the health consequence of dietary diversity and sociodemographic disparities in dietary diversity, but the temporal change of dietary diversity has been less frequently discussed (22). Some studies have documented that the chewing and digestive function of older adults will gradually decline with age, thereby limiting their dietary diversity (22, 23). However, as time goes by, the macro-socioeconomic level, agricultural industry developmental level, and food richness will also gradually increase, thus contributing to the improvement of dietary diversity (24). In addition, prior studies have reported that higher SES can offset the declining trend of dietary diversity with age more or less (19). However, few studies have examined the cohort change in dietary diversity. The nutrition intake and dietary health of older adults born before the 1950s, when economic development was lagging behind, have experienced profound changes in the life stage of middle-aged and older for decades, and it is highly related to the rapid socioeconomic change of China and the life course of older adults, as well as their combination, in recent years (8, 22). In addition, effects of rural–urban residence and SES on dietary diversity among older adults may also vary with age and across successive cohorts. For example, with the improvement of living conditions of older adults in rural areas and with low and middle SES, the positive effect of high SES and urban residence on dietary health may weaken, which still needs further empirical research (25).

China has been experiencing an urban–rural dual-track system for a long time, with obvious disparities in socioeconomic development between urban and rural areas (26). Urban–rural residence and SES are important influencing factors of DDS among Chinese older adults (19, 21). Accordingly, investigating the age and cohort effects of DDS or dietary health, as well as their urban–rural and SES disparities, can help comprehensively understand the temporal change of DDS in Chinese older adults, master the influence of social change and individual life course on dietary health, and grasp the dynamic impact of SES and urban–rural residence on older adults’ DDS from the life course perspective. These will further help identify specific social contexts and life course nodes in which residence and SES play a role in promoting DDS and dietary health, and help perform targeted dietary and nutrition intake interventions for Chinese older adults to achieve healthy aging. Therefore, based on the Chinese Longitudinal Healthy Longevity Survey (CLHLS) data from 2002 to 2018, this study attempted to explore the temporal change in DDS among Chinese older adults and the SES heterogeneity in such change from the perspective of urban–rural differentiation.

## Materials and methods

### Data source

**FIGURE 1:**
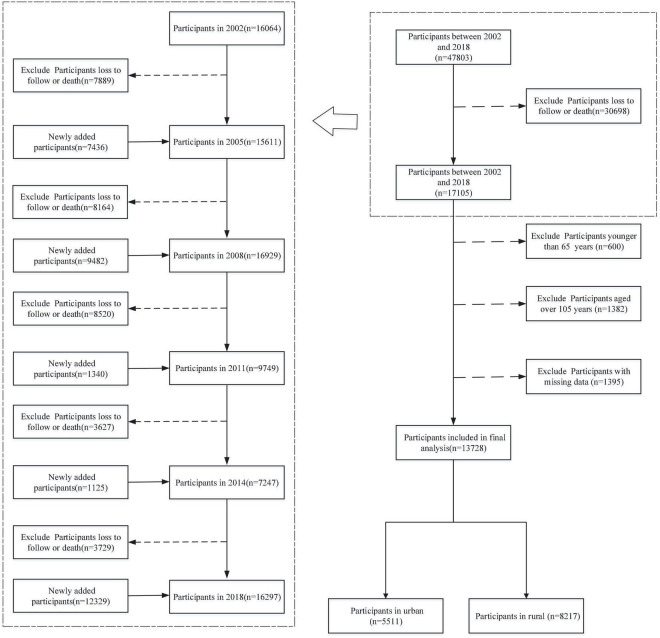
Flowchart of sample selection.

The data used in this study were obtained from the CLHLS, which started in 1998 and with follow-up surveys in 2000, 2002, 2005, 2008, 2011, 2014, and 2018. Participants in the CLHLS of 1998 and 2000 were limited to those aged 80 years or older, and those aged 65–79 years were added to the 2002 survey and subsequent waves, so only CLHLS data from 2002 to 2018 were used in this study. The survey involved samples from 23 provinces, municipalities, or autonomous regions in China in which 85% of the total Chinese population resides, which has been verified to be accurate, reliable, and representative. Using a structured questionnaire, trained interviewers carried out the survey at participants’ homes. The disabled oldest-old agreed to participate through proxy assistance by proxy respondents (usually their spouse or other close family members) if they were unable to independently answer the questionnaire. Details about the CLHLS have been described elsewhere (27). In the present study, only participants aged between 65 and 105 years were included, and we excluded participants who failed to report the variables used (response rate = 92.3%). A total of 13,728 participants were included in final analysis (see Figure 1). The CLHLS program was approved by the Research Ethics Committee of Peking University (IRB00001052-13074), and written informed consent was provided by all participants or their proxy respondents; therefore, this secondary analysis using CLHLS data did not require additional ethical approval.

### Variables

#### Dietary diversity

According to previous studies (11, 28–30), this study applied DDS to evaluate older adults’ dietary diversity. Specific intake frequencies and scoring criteria are listed in Table 1. DDS was used as a continuous variable, with a score range of 0–8 in which 8 represented the highest level of dietary diversity.

**TABLE 1:** Specific intake frequency and scoring criteria.

Food group	DDS
Fresh fruits Fresh vegetables	“Every day/almost every day” = 1 “Often” = 1 “Sometimes” = 0 “Rarely or never” = 0
Meat Fish Eggs Bean products Garlic Tea	“Almost every day” = 1 “At least once a week” = 1 “At least once a month” = 0 “Occasionally” = 0 “Rarely or never” = 0

#### Socioeconomic status

Education, family income, and perceived or self-reported income status were used as SES measures in this study. For education, respondents were asked “How many years did you attend school?” Due to the large number of respondents who answered 0, education was treated as a binary variable, with “0” indicating illiteracy and “1” indicating literacy. Family income was adjusted on the basis of 2010 constant price. Then, we used the natural logarithm of adjusted family income as one of the elements of SES, where a higher value indicated higher SES. In this questionnaire, the respondents were asked “How about your life compared with other local people?” We coded “very poor” as 1, “poorer” as 2, “fair” as 3, “richer” as 4, and “very rich” as 5 to measure perceived income status.

#### Age and cohort

Age was a time-varying variable ranging from 65 to 105. The birth year ranged from 1916 to 1949, and it was recoded as four dummy variables, namely, cohort1916, cohort1926, cohort1936, and cohort1946. Using 10-years as an interval, participants born from 1916 to 1925 were included in cohort1916, cohort1926 and cohort1936 were coded in the same way, and participants born from 1946 to 1949 were included in cohort1946.

#### Covariates

Based on previous studies (9, 21, 31), several demographic factors were used as control variables. Gender was measured dichotomously, with “1” coding female and “0” male. Current marital status was measured dichotomously, with “1” coding for people who were currently married and living with spouse and “0” for others. Former occupation was measured by the occupation held before they were 60 years old, and the categories of occupation in the questionnaire included professional and technical personnel, governmental, institutional or managerial personnel, commercial, service or industrial worker, self-employed worker, farmer, house worker, and others. In this study, the professional and technical personnel, governmental, institutional or managerial personnel, commercial, service or industrial worker, and self-employed worker were recoded as formal occupation “1,” and others were recoded as unformal occupation “0.” Self-reported health (SRH) was assessed on a 5-point Likert scale item ranging from 1 (very poor) to 5 (excellent). Multimorbidity was measured as a composite indicator of the sum of 13 chronic diseases or conditions, and details are described in Supplementary File P1.

### Statistical analysis

Participants were grouped into urban and rural groups according to the place of residence. To examine the association between SES and DDS, we performed a series of linear mixed models, with the unadjusted model including SES, residence, age, and cohort, and the adjusted model including SES, residence, age, cohort, gender, marital status, former occupation, multimorbidity, and SRH. Finally, we explored trends of the association between SES and DDS with age and across successive cohorts by including their interaction items in the model, and these models were estimated separated by residence (urban and rural). The final two-level mixed-effects linear regression model with random intercepts was defined as follows:

$$\begin{array}{l} DDS_{ij}={\text{π}}_{0}+{\text{π}}_{1}*\;age_{ji}+{\text{π}}_{2}*\;age_{ij}^{2}+{\text{π}}_{3}*\;SES_{ij}{\text{+π}}_{4}\\ {}*\;cohort_{j}+{\text{π}}_{5}*\;age_{ij}*\;SES_{ij}+{\text{π}}_{6}*\;cohort_{j}*\;SES_{ij}\\ +{\text{π}}_{7}*\;age_{ij}*\;cohort_{j}*\;SES_{ij}+\sum {\text{π}}_{p}*\;covariates_{ij}\\ +\sum {\text{π}}_{p}*\;covariates_{i}+{\text{μ}}_{0j}+{\text{ε}}_{ij} \end{array}$$

where *DDS*_*ij*_ denotes DDS for an individual *j* in a period *i*; π_0_ is the time-invariant fixed intercept; π_1_ to π_7_ are coefficients of age, age square, SES, cohort, and their interaction items; π_*p*_ and π_*q*_ are coefficients of covariates being time-varying and time-invariant; μ_0*j*_ is the random intercept that is specific to each individual, and ε_*ij*_ is the residual for an individual *j* in a period *i*.

All statistical analyses were performed using SAS version 9.4 (SAS Institute Inc., Cary, NC, USA). The restricted maximum likelihood estimation in SAS procedure “GLIMMIX” was used. All *p*-values were two-sided, and statistical significance was defined as *p* < 0.05.

## Empirical results

### Descriptive statistics

A total of 13,728 older adults (rural 59.86% and urban 40.14%) with completed data were included at the baseline. Participants’ main baseline characteristics are presented in Table 2.

**TABLE 2:** Baseline information of dietary diversity score (DDS), sociodemographic factors, and health.

	Rural (*n* = 8,217)		Urban (*n* = 5,511)	
	Mean/n	SD/%	Mean/n	SD/%
**DDS**	4.06	2.24	4.64	2.19
**Education**				
Illiteracy	4193	51.03	2263	41.06
Literacy	4024	48.97	3248	58.94
**Perceived income status**				
Very poor	219	2.67	103	1.87
Poorer	1184	14.41	578	10.49
Fair	5621	68.41	3736	67.79
Richer	1123	13.67	997	18.09
Very rich	70	0.85	97	1.76
**Family income**	8.20	1.38	8.77	1.26
**Age**	80.77	10.09	80.76	9.79
**Cohort**				
Cohort1916	1318	23.92	1834	22.32
Cohort1926	1878	34.08	2632	32.03
Cohort1936	1500	27.22	2366	28.79
Cohort1946	815	14.79	1385	16.86
**Gender**				
Male	3960	48.19	2667	48.39
Female	4257	51.81	2844	51.61
**Marital status**				
Married and living with spouse	4648	56.57	3019	54.78
Others	3569	43.43	2492	45.22
**Former occupation**				
Formal	1037	12.62	2800	50.81
Unformal or others	7180	87.38	2711	49.19
**Multimorbidity**	1.53	1.17	1.64	1.30
**SRH**	2.50	0.87	2.56	0.89

SD, standard deviation; DDS, dietary diversity score; SRH, self-reported health.

In rural China, the participants were more like to be male, unlettered, not currently married or live with spouse, and have no formal occupation before 60. The average DDS of the rural participants was 4.06, the average perceived income status was 2.96, the average natural logarithm of adjusted family income was 8.20, the average age was 80.77, and the proportion of participants in cohort1916, cohort1926, cohort1936, and cohort1946 was 23.92%, 34.08%, 27.22%, and 14.79%, respectively. The average multimorbidity of the rural participants was 1.53, and the average SRH score was 2.50.

In urban China, the participants were more like to be female, unlettered, not currently married or live with spouse, and have former occupation. The average DDS of the urban participants was 4.64, the average perceived income status was 3.07, the average natural logarithm of adjusted family income was 8.77, the average age was 80.76, and the proportion of participants in cohort1916, cohort1926, cohort1936, and cohort1946 was 22.32%, 32.03%, 29.79%, and 16.86%, respectively. The average multimorbidity of the urban participants was 1.53, and the average SRH score was 2.56.

### Association between socioeconomic status and dietary diversity score

Table 3 shows linear mixed regression results of the association between SES and DDS in Chinese older adults. With covariates adjusted for, the goodness of fit of the model became better (both −2LL and BIC). Although coefficients of SES slightly changed, they remained statistically significant. Overall, SES was positively associated with DDS in both rural and urban areas. In the adjusted model, coefficients of education were 0.1645 (*p* = 0.0003) and 0.0932 (*p* = 0.0080) in urban and rural areas, respectively; coefficients of perceived income status were 0.2638 (*p* < 0.0001) and 0.4063 (*p* < 0.0001) in urban and rural areas, respectively; and coefficients of family income were 0.2917 (*p* < 0.0001) and 0.2421 (*p* < 0.0001) in urban and rural areas, respectively.

**TABLE 3:** Association between age, cohort, and dietary diversity score (DDS) in Chinese older adults.

	Model 1: urban			Model 2: rural			Model 3: urban			Model 4: rural		
	β	SE	*p*	β	SE	*p*	β	SE	*p*	β	SE	*p*
**Fixed effect**												
Intercept	0.8161	0.1439	< 0.0001	0.6121	0.1043	< 0.0001	0.6859	0.1703	< 0.0001	0.3050	0.1269	0.0163
Education (ref =illiteracy)	0.4403	0.0415	< 0.0001	0.2540	0.0332	< 0.0001	0.1645	0.0452	0.0003	0.0932	0.0351	0.0080
Perceived income status	0.3747	0.0264	< 0.0001	0.5152	0.0212	< 0.0001	0.2638	0.0268	< 0.0001	0.4063	0.0217	< 0.0001
Family income	0.3232	0.0137	< 0.0001	0.2462	0.0099	< 0.0001	0.2917	0.0138	< 0.0001	0.2421	0.0099	< 0.0001
Age	0.0026	0.0086	0.7585	0.0444	0.0065	< 0.0001	0.0189	0.0086	0.0276	0.0559	0.0066	< 0.0001
Age square	0.0008	0.0002	0.0003	0.0002	0.0002	0.2879	0.0007	0.0002	0.0008	0.0001	0.0002	0.3740
Cohort (ref = cohort1946)												
Cohort1916	−1.2671	0.1145	< 0.0001	−1.9532	0.0915	< 0.0001	−1.5219	0.1141	< 0.0001	−2.0926	0.0909	< 0.0001
Cohort1926	−0.9187	0.0897	< 0.0001	−1.2914	0.0693	< 0.0001	−1.0920	0.0889	< 0.0001	−1.3749	0.0687	< 0.0001
Cohort1936	−0.4188	0.0675	< 0.0001	−0.6360	0.0524	< 0.0001	−0.5200	0.0661	< 0.0001	−0.6680	0.0512	< 0.0001
Control variables	no			no			yes			yes		
**Random effect**												
Level-1: within-person	3.5869	0.0496	< 0.0001	3.5901	0.0394	< 0.0001	3.5502	0.0490	< 0.0001	3.5779	0.0392	< 0.0001
Level-2: between-person	0.6670	0.0409	< 0.0001	0.7502	0.0338	< 0.0001	0.5731	0.0386	< 0.0001	0.6461	0.0323	< 0.0001
**Model fit**												
−2LL	67539			106522			67146			106026		
BIC	67556			106540			67163			106044		

β, coefficient; SE, standard error; −2LL, −2 log likelihood; BIC, Bayesian Information Criterion.

### Association between age, cohort, and dietary diversity score

Table 3 also presents the association between age, cohort, and DDS in Chinese older adults. With covariates adjusted for, the goodness of fit of the model became better (both −2LL and BIC). Although coefficients of cohort slightly changed, they remained statistically significant. By contrast, the coefficient of age was not statistically significant in Model 1, but it became significant after controlling for covariates in Model 3. Specifically, age was positively associated with DDS in both urban and rural areas. Coefficients of age and age square were 0.0189 (*p* = 0.0276) and 0.0007 (*p* = 0.0008) in urban areas, whereas they were 0.0559 (*p* < 0.0001) and 0.0001 (*p* = 0.3740) in rural areas.

The association between cohort and DDS was also statistically significant in both urban and rural areas. In urban areas, compared with older adults born in cohort1946, those born earlier had a lower DDS (for cohort1916: β = −1.5219, *p* < 0.0001; for cohort1926: β = −1.0920, *p* < 0.0001; for cohort1936: β = −0.5200, *p* < 0.0001). In rural areas, compared with older adults born in cohort1946, those born earlier also had a lower DDS (for cohort1916: β = −2.0926, *p* < 0.0001; for cohort1926: β = −1.3749, *p* < 0.0001; for cohort1936: β = −0.6680, *p* < 0.0001).

### Association between socioeconomic status, age, cohort, and dietary diversity score

We further assessed potential modification effects between SES and age/cohort on DDS. Supplementary Tables 1–4 report the model results after including the interaction term of SES and age and cohort in both urban and rural areas, respectively. Significant moderating effects were observed for age and cohort in both urban and rural areas. Supplementary Figures 1–3 present estimated DDS trajectories across different SES groups that changed with age and across successive cohorts in urban and rural areas. Supplementary Tables 5, 6 present the results of three-way interaction effects on DDS, including education–age–cohort, family income–age–cohort, and perceived income status–age–cohort, in Chinese older adults.

It is observed that the effect of SES on older adults’ DDS varied with age and across successive cohorts. In urban areas, the main effect of education on DDS was significantly positive (β = 0.8654, *p* < 0.0001), and this effect would reduce with an increase in age (β = −0.0622, *p* < 0.0001), but it would slightly increase across successive cohorts (cohort1946 as reference, for cohort1916: β = −1.0332, *p* < 0.0570). When it comes to family income and perceived income status, the results were similar (see Supplementary Table 5 or Supplementary Figures 2, 3). Also, age and cohort trajectories of the effect of SES on rural older adults’ DDS were also highly similar to those in urban older adults, and the SES disparities in age and cohort trajectories were even larger, so we did not further report the results in Supplementary Table 6 (also see Supplementary Figures 1–3).

**FIGURE 2:**
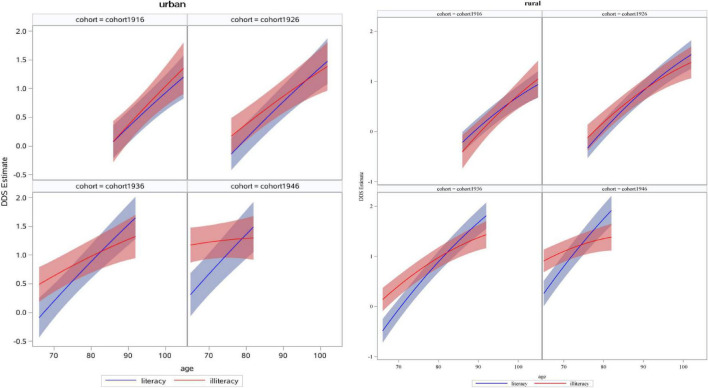
Estimated dietary diversity score (DDS) of education with age and across successive cohorts in urban and rural older adults.

Figure 2 presents the age trajectory of DDS at different educational levels and across successive cohorts in urban and rural older adults (also see Supplementary Tables 5, 6). In urban areas, the educational disparity in DDS among older adults remained unchanged with age in cohort1916 and cohort1926, while it narrowed in cohort1936 and cohort1946. The age trajectory across successive cohorts in rural areas were highly similar to those in urban areas.

**FIGURE 3:**
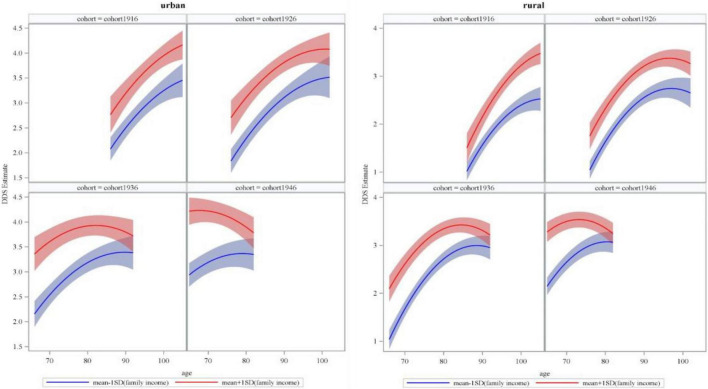
Estimated dietary diversity score (DDS) of family income with age and across successive cohorts in urban and rural older adults.

Figure 3 presents the age trajectory of DDS at different family income levels (1 standard deviation above the mean, 1 standard deviation below the mean) and across successive cohorts in urban and rural older adults (also see Supplementary Tables 5, 6). In urban areas, the disparity in DDS between older adults with more and less family income remained unchanged with age in cohort1916 and cohort1926, but it narrowed in cohort1936 and cohort1946. In rural areas, the disparity in DDS between older adults with more and less family income enlarged with age in cohort1916, remained unchanged in cohort1926, and narrowed in cohort1936 and cohort1946.

**FIGURE 4:**
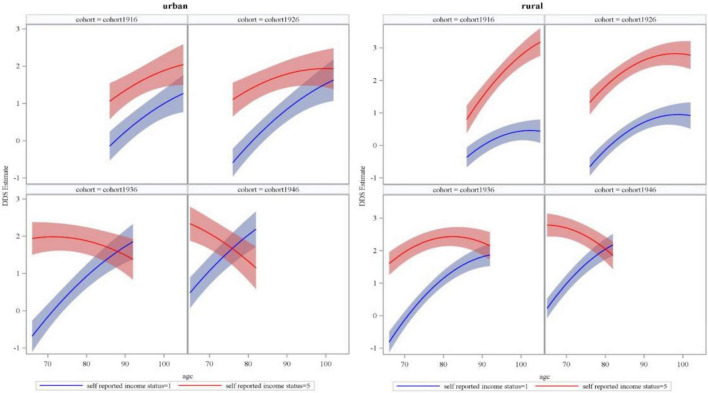
Estimated dietary diversity score (DDS) of perceived or self-reported income status with age and across successive cohorts in urban and rural older adults.

Figure 4 presents the age trajectory of DDS at different perceived income levels (1 and 5) and across successive cohorts in urban and rural older adults (also see Supplementary Tables 5, 6). In urban areas, the disparity in DDS between older adults with higher and lower perceived income status enlarged with age in cohort1916, remained unchanged in cohort1926, and significantly narrowed in cohort1936 and cohort1946. In rural areas, the disparity in DDS between older adults with higher and lower perceived income status remained unchanged in cohort1916, but it significantly narrowed with age in cohort1926, cohort1936, and cohort1946.

## Discussion

Increasing dietary diversity is an important intervention for older adults to maintain dietary nutrition and physical and mental health (6, 8, 12), and SES is an important determinant of dietary diversity in Chinese older adults (17, 19). In the context of rapid social transition and development, this study re-examined the effect of urban–rural residence and SES on dietary diversity among older adults in China and further explored variations of the effect with age and across successive cohorts from the life course perspective. We found that older adults living in urban areas and with higher SES generally had higher DDS, but the positive effect of urban residence on DDS decreased with age and increased across successive cohorts. The positive effect of urban residence and higher SES on DDS in more recent cohorts gradually weakened or even disappeared with an increase in age. However, for the earliest cohort, the effect of SES on DDS was complex, where education did not play a significant role, whereas the positive effects of family income and perceived economic status slightly increased with age or remained unchanged.

Residence and SES still had robust positive effects on dietary diversity in Chinese older adults. Living in an urban area, more education, a higher level of family income, and perceived economic status were all associated with higher DDS, which is highly consistent with the findings in many developing countries (17, 19), but different from those in developed countries (12, 13, 20). Compared with developed countries, the dietary diversity of older adults in developing countries is more likely to be positively influenced by high SES because the marginal benefit of SES is greater in developing countries, and people living in developing countries usually experience and live in environments where SES gains are more unequal (17). Urban areas have better food markets than rural areas, so the former has higher food accessibility and diversity (21, 32). Furthermore, higher SES can not only ensure individuals to have stronger food purchasing power but also help them own higher awareness of dietary health and cultivate good eating habits (33, 34).

The DDS of Chinese older adults would gradually increase with age and across successive cohorts. Many studies have shown that the dietary diversity of older adults decreases with age because the decline of chewing and digestive functions caused by aging will lead to lower dietary diversity (22). However, this study found that due to the rapid socioeconomic development and prosperity of the food market in current China (33), the dietary diversity of urban and rural older adults has increased with age in recent years. Older adults born later can enjoy more benefits and diversified diets brought about by social development, suggesting that the dietary diversity increase brought about by socioeconomic development can compensate for the decline in dietary diversity caused by physiological aging. Although the DDS of urban older adults was significantly higher than that of their rural counterparts as a whole, this gap narrowed with age, and there was no significant difference in older adults older than 80 years. This may be related to social development policies in China such as the poverty eradication strategy. The government has more basic social welfare policies for the oldest-old with less income in rural areas to ensure their healthy diet (35). By contrast, the DDS gap between urban and rural older adults gradually widened across successive cohorts. A possible explanation is that China has focused on urban development for quite a long time, so there is a large socioeconomic gap between urban and rural areas (36), making rural older adults born later experience more unfortunate life events in their early life and own worse body function in their old age than their urban counterparts (37), thus limiting the increase in dietary diversity.

Education had no significant effect on the DDS of older adults born earlier but had a positive effect on the DDS of those born later. However, this effect would gradually decrease with age, and there was no significant effect in the oldest-old born later. On the one hand, education has developed significantly in the early days of new China, which enables older adults born later to receive more and higher quality education (38, 39) and highlights the significance of education in health awareness and healthy eating behavior (40, 41). On the other hand, due to the health and survival selection bias, the dietary diversity of the oldest-old may no longer depend on the health awareness and literacy brought about by education, but on personal physique, social network support, family economic conditions, and national social security policies, which is similar to the finding of Xu and Jiang’s study (42). They found that the positive effect of cultural capital such as education on various health behaviors of older adults would decrease with age, while the effect of social capital would gradually increase.

Temporal trajectories of the influence of subjective and objective economic conditions on the DDS of older adults were inconsistent. Objectively, the positive effect of family income on DDS increased with age in earlier cohorts but decreased in more recent cohorts and almost disappeared in the oldest-old. Subjectively, compared with urban samples in earlier cohorts, perceived economic status had a stronger positive effect on the DDS of rural samples, and this effect significantly increased with age. However, the effect of perceived economic status on the DDS of urban and rural older adults in more recent cohorts weakened with age, and this effect almost disappeared in the oldest-old (similar results were observed for family income). Family income and perceived economic status are direct reflections of economic capital and play similar roles with education in more recent cohorts. Previous studies have documented that the positive impact of economic capital represented by family income on various health behaviors of older adults in China weakens with age (42), which is consistent with our results. Economic status at the subjective level usually treats surrounding people as the reference (43). The living environment of rural older adults is more traditional, especially for the oldest-old who were born earlier. Their evaluation on economic status is more likely to be based on the traditional culture of “having ample food and clothing,” rather than emulative money comparison (44). In addition, when the absolute material condition is poorer than that of their urban counterparts in the same period, there may be a stronger health and survival selection bias among rural older adults born earlier, for whom living longer depends on and reflects healthy eating behaviors more. These phenomena reinforce the positive effect of subjective economic status on DDS among rural oldest-old adults in China.

These results have important insights for intervention of dietary health in Chinese older adults. On the one hand, we should not only examine the dietary health problem of older adults from the perspective of age but also pay attention to the role of birth cohort. For older adults who experienced a special historical period in the early 20th century, especially the oldest-old in rural China, more attention should be paid to their dietary health problem to help them fully enjoy the achievement of social development. On the other hand, it is necessary to pay attention to the dietary diversity disadvantage of older adults with low SES. However, the dietary diversity advantage brought about by SES among older adults at present may gradually disappear with age because the promotion effect of SES on dietary diversity is long-lasting and occurs across the life course. Therefore, we cannot rely too much on improving SES to make up for the lack of dietary diversity. By contrast, we need to make efforts from social networks, community living environments, and social welfare policies to improve the dietary health of older adults when they were still young.

Based on the long-term, nationally representative large sample follow-up data, this study re-examined the effect of rural–urban residence and SES on the dietary diversity of older adults from the perspective of life course, contributing new knowledge on the joint effect of micro-individual and macro-society on dietary health among older adults. Nevertheless, this study still has some limitations. First, this study used eight kinds of food to measure dietary diversity as it was hard to cover all food types and capture specific dietary tendencies of older adults (e.g., whether they were vegetarian or meat-based). Second, age and cohort trajectories of Chinese older adults’ DDS implied historical details on how social transition affected individual health behavior. However, this study simply presented these trajectories and failed to link them to specific characteristics of social transition, making the study less convincing. These issues need to be discussed in subsequent studies.

## Conclusion

The dietary diversity of older adults in China was affected by urban–rural residence and SES, but these effects changed with age and across successive cohorts. The advantage of dietary diversity brought about by urban residence would decrease with age and increase across successive cohorts. The positive effect of urban residence and higher SES on DDS among older adults in more recent cohorts would gradually weaken or even disappear with age. However, for older adults in the earliest cohort, the effect of SES on dietary diversity was complex, where education did not play a significant role, whereas the positive effect of family income and perceived economic status would slightly increase with age or remain unchanged.

## Data availability statement

The original contributions presented in this study are included in the article/Supplementary material, further inquiries can be directed to the corresponding author.

## Author contributions

JJ and NC: conceptualization and methodology. YY, NC, and JJ: writing—original draft preparation. YY, JJ, and AH: writing—review and editing. NC: visualization. All authors have read and agreed to the published version of the manuscript.
