# Supplementary material for: Age and cohort trends of the impact of socioeconomic status on dietary diversity among Chinese older adults from the perspective of urban–rural differences: A prospective cohort study based on CLHLS 2002–2018
Source: Front Nutr. 2022 Oct 20;9:1020364. doi: 10.3389/fnut.2022.1020364 (PMC9632445; doi:10.3389/fnut.2022.1020364)
Supplement: Supplementary file 1 [file Data_Sheet_1.docx]

**Contents**

[The measurement for multimorbidity 1](#_Toc109816627)

[Table S1 Associations between SES, age and older adults’ DDS in urban China 2](#_Toc109816628)

[Table S2 Associations between SES, age and older adults’ DDS in rural China 3](#_Toc109816629)

[Table S3 Associations between SES, cohort and older adults’ DDS in urban China 4](#_Toc109816630)

[Table S4 Associations between SES, cohort and older adults’ DDS in rural China 5](#_Toc109816631)

[Table S5 Associations between SES, age, cohort and older adults’ DDS in urban China 6](#_Toc109816632)

[Table S6 Associations between SES, age, cohort and older adults’ DDS in rural China 8](#_Toc109816633)

[Figure S1 Estimated DDS of education with age and across successive cohorts in urban and rural older adults 10](#_Toc109816634)

[Figure S2 Estimated DDS of percieved or self-reported income status with age and acorss successive cohorts in urban and rural older adults 11](#_Toc109816635)

[Figure S3 Estimated DDS of family income with age and acorss successive cohorts in urban and rural older adults 12](#_Toc109816636)

[Figure S4 Normality test for models’ residuals using Q-Q plots 13](#_Toc109816636)

# The measurement for multimorbidity

Nine self-reported chronic conditions were ascertained by asking participants whether they had the following chronic conditions: diabetes, heart disease, cerebrovascular disease, cancer, respiratory diseases (bronchitis, emphysema, asthma, and pneumonia), gastric or duodenal ulcer, Parkinson’s disease, arthritis, and prostate or gynecological diseases. Blood pressure was measured by a trained physician with the electronic sphygmomanometer (Omron HEM-7200 Monitor), and the mean of two repeated measures was calculated. Hypertension was defined as a self-reported condition or systolic blood pressure ≥140 mmHg or diastolic blood pressure ≥90 mmHg. Cognitive impairment was defined as having self-reported dementia and (or) poor cognitive function. Cognitive function was measured by the Chinese version of the Mini-Mental State Examination (MMSE) during each survey. The validity and reliability of the Chinese MMSE have been verified previously. The MMSE score ranged from 0 to 30, and poor cognitive function was defined as the MMSE score ≤23. Participants were considered to have vision impairment if they could not see or distinguish the break in the circle or were blind and/or reported having either glaucoma or cataract. Hearing loss was defined as requiring a hearing aid when listening.

| Table S1 Associations between SES, age and older adults’ DDS in urban China | | | | | | | | | | | | | | | | |
| --- | --- | --- | --- | --- | --- | --- | --- | --- | --- | --- | --- | --- | --- | --- | --- | --- |
|  | Model s1 | | | |  |  | Model s2 | | | | |  |  | Model s3 | | |
|  | *β* | | SE | *p* |  |  | *β* | | SE | | *p* |  |  | *β* | SE | *p* |
| **fixed effect** | | | | |  | **fixed effect** | | | | | |  | **fixed effect** | | | |
| intercept | | 0.4410 | 0.1827 | 0.0158 |  | intercept | 0.3567 | | 0.2259 | | 0.1144 |  | intercept | -0.3479 | 0.2842 | 0.2208 |
| education (ref=illiteracy) | | | | |  | education (ref=illiteracy) | | | | | |  | education (ref=illiteracy) | | | |
|  | | 0.4755 | 0.0957 | <.0001 |  |  | 0.1651 | | 0.0452 | | 0.0003 |  |  | 0.1617 | 0.0451 | 0.0003 |
| age | | 0.0346 | 0.0096 | 0.0003 |  | perceived income | 0.3736 | | 0.0564 | | <.0001 |  | perceived income | 0.2639 | 0.0267 | <.0001 |
| age*education | | -0.0159 | 0.0043 | 0.0002 |  | age | 0.0376 | | 0.0121 | | 0.0018 |  | family income | 0.4098 | 0.0295 | <.0001 |
| perceived income | | 0.2643 | 0.0267 | <.0001 |  | perceived income *age | -0.0060 | | 0.0027 | | 0.0272 |  | age | 0.0643 | 0.0132 | <.0001 |
| family income | | 0.2897 | 0.0138 | <.0001 |  | family income | 0.2916 | | 0.0138 | | <.0001 |  | family income*age | -0.0064 | 0.0014 | <.0001 |
| age square | | 0.0006 | 0.0002 | 0.0090 |  | age square | 0.0007 | | 0.0002 | | 0.0008 |  | age square | 0.0011 | 0.0002 | <.0001 |
| cohort (ref=cohort1946) | | | | |  | cohort (ref=cohort1946) | | | | | |  | cohort (ref=cohort1946) | | | |
| cohort1916 | | -1.5154 | 0.1140 | <.0001 |  | cohort1916 | -1.5249 | | 0.1141 | | <.0001 |  | cohort1916 | -1.5027 | 0.1140 | <.0001 |
| cohort1926 | | -1.0686 | 0.0891 | <.0001 |  | cohort1926 | -1.0938 | | 0.0889 | | <.0001 |  | cohort1926 | -1.0120 | 0.0905 | <.0001 |
| cohort1936 | | -0.4940 | 0.0665 | <.0001 |  | cohort1936 | -0.5205 | | 0.0661 | | <.0001 |  | cohort1936 | -0.4443 | 0.0681 | <.0001 |
| control variables | | yes |  |  |  | control variables | yes | |  | |  |  | control variables | yes |  |  |
| **random effect** | |  |  |  |  | **random effect** | | | | | |  | **random effect** | | | |
| level-1: within-person | | 3.5482 | 0.0489 | <.0001 |  | level-1: within-person | 3.5513 | 0.0490 | | <.0001 | |  | level-1: within-person | 3.5500 | 0.0490 | <.0001 |
| level-2: between-person | | 0.5717 | 0.0386 | <.0001 |  | level-2: between-person | 0.5704 | 0.0386 | | <.0001 | |  | level-2: between-person | 0.5668 | 0.0386 | <.0001 |
| **model fit** | | | | |  | **model fit** |  | | | | |  | **model fit** |  | | |
| -2LL | | 67141 | | |  | -2LL | 67151 | | | | |  | -2LL | 67137 | | |
| BIC | | 67159 | | |  | BIC | 67168 | | | | |  | BIC | 67154 | | |

| Table S2 Associations between SES, age and older adults’ DDS in rural China | | | | | | | | | | | | | | | | |
| --- | --- | --- | --- | --- | --- | --- | --- | --- | --- | --- | --- | --- | --- | --- | --- | --- |
|  | Model s1 | | | |  |  | | Model s2 | | |  |  | | Model s3 | | |
|  | *β* | SE | | *p* |  |  |  | *β* | SE | *p* |  |  |  | *β* | SE | *p* |
| **fixed effect** | | | | |  | **fixed effect** | | | | |  | **fixed effect** | | | | |
| intercept | 0.0799 | | 0.1337 | 0.5504 |  | intercept | | -0.3969 | 0.1710 | 0.0203 |  | intercept | | -0.2163 | 0.1986 | 0.2761 |
| education (ref=illiteracy) | | | | |  | education (ref=illiteracy) | | | | |  | education (ref=illiteracy) | | | | |
|  | 0.4151 | 0.0697 | | <.0001 |  |  | | 0.0932 | 0.0351 | 0.0080 |  |  | | 0.0913 | 0.0351 | 0.0093 |
| age | 0.0704 | 0.0071 | | <.0001 |  | perceived income | | 0.3750 | 0.0446 | <.0001 |  | perceived income | | 0.4078 | 0.0217 | <.0001 |
| age*education | -0.0177 | 0.0033 | | <.0001 |  | age | | 0.0508 | 0.0090 | <.0001 |  | family income | | 0.3053 | 0.0210 | <.0001 |
| perceived income | 0.4079 | 0.0217 | | <.0001 |  | perceived income*age | | 0.0017 | 0.0022 | 0.4222 |  | age | | 0.0784 | 0.0093 | <.0001 |
| family income | 0.2401 | 0.0099 | | <.0001 |  | family income | | 0.2420 | 0.0099 | <.0001 |  | family income*age | | -0.0035 | 0.0010 | 0.0006 |
| age square | 0.0000 | 0.0002 | | 0.9077 |  | age square | | 0.0001 | 0.0002 | 0.3854 |  | age square | | 0.0004 | 0.0002 | 0.0415 |
| cohort (ref=cohort1946) | | | | |  | cohort (ref=cohort1946) | | | | |  | cohort (ref=cohort1946) | | | | |
| cohort1916 | -2.1112 | 0.0909 | | <.0001 |  | cohort1916 | | -2.0913 | 0.0909 | <.0001 |  | cohort1916 | | -2.0896 | 0.0909 | <.0001 |
| cohort1926 | -1.3530 | 0.0688 | | <.0001 |  | cohort1926 | | -1.3749 | 0.0687 | <.0001 |  | cohort1926 | | -1.3357 | 0.0696 | <.0001 |
| cohort1936 | -0.6345 | 0.0516 | | <.0001 |  | cohort1936 | | -0.6688 | 0.0512 | <.0001 |  | cohort1936 | | -0.6272 | 0.0526 | <.0001 |
| control variables | yes | | | |  | control variables | | yes | | |  | control variables | | yes | | |
| **random effect** | | | | |  | **random effect** | | | | |  | **random effect** | | | | |
| level-1: within-person | 3.5729 | 0.0392 | | <.0001 |  | level-1: within-person | | 3.5779 | 0.0392 | <.0001 |  | level-1: within-person | | 3.5769 | 0.0392 | <.0001 |
| level-2: between-person | 0.6468 | 0.0323 | | <.0001 |  | level-2: between-person | | 0.6462 | 0.0323 | <.0001 |  | level-2: between-person | | 0.6451 | 0.0322 | <.0001 |
| **model fit** | | | | |  | **model fit** | | | | |  | **model fit** | | | | |
| -2LL | 106007 | | | |  | -2LL | 106036 | | | |  | -2LL | 106026 | | | |
| BIC | 106025 | | | |  | BIC | 106045 | | | |  | BIC | 106044 | | | |

| Table S3 Associations between SES, cohort and older adults’ DDS in urban China | | | | | | | | | | | | | | | | | | | |
| --- | --- | --- | --- | --- | --- | --- | --- | --- | --- | --- | --- | --- | --- | --- | --- | --- | --- | --- | --- |
|  | | Model s1 | | | |  | Model s2 | | | | | |  | Model s3 | | | | | |
|  |  | *β* | SE | | *p* |  |  | *β* | | | SE | *p* |  |  | *β* | SE | | *p* | |
| **fixed effect** | | | | | |  | **fixed effect** | | | | | |  | **fixed effect** | | | | | |
| intercept | 0.4061 | | 0.2015 | | 0.0438 |  | intercept | 0.9607 | | | 0.2512 | 0.0001 |  | intercept | 0.3880 | 0.3221 | | 0.2284 | |
| education (ref=illiteracy) | | | | | |  | education (ref=illiteracy) | | | | | |  | education (ref=illiteracy) | | | | | |
|  | 0.5098 | | 0.1388 | | 0.0002 |  |  | 0.1644 | | | 0.0452 | 0.0003 |  |  | 0.1641 | 0.0452 | | 0.0003 | |
| perceived income | 0.2631 | | 0.0268 | | <.0001 |  | perceived income | 0.1753 | | | 0.0660 | 0.0080 |  | perceived income | 0.2637 | 0.0268 | | <.0001 | |
| family income | 0.2908 | | 0.0138 | | <.0001 |  | family income | 0.2916 | | | 0.0138 | <.0001 |  | family income | 0.3249 | 0.0324 | | <.0001 | |
| cohort (ref=cohort1946) | | | | | |  | cohort (ref=cohort1946) | | | | | |  | cohort (ref=cohort1946) | | | | | |
| cohort1916 | -1.1770 | | 0.1687 | | <.0001 |  | cohort1916 | | -1.7538 | | 0.2905 | <.0001 |  | cohort1916 | -0.9538 | 0.4239 | | 0.0244 | |
| cohort1926 | -0.7737 | | 0.1528 | | <.0001 |  | cohort1926 | -1.3943 | | | 0.2588 | <.0001 |  | cohort1926 | -0.7290 | 0.3818 | | | 0.0562 |
| cohort1936 | -0.2271 | | 0.1465 | | 0.1210 |  | cohort1936 | -0.9115 | | | 0.2513 | 0.0003 |  | cohort1936 | -0.2317 | 0.3664 | | 0.5273 | |
| education* cohort1916 | -0.4418 | | 0.1599 | | 0.0057 |  | perceived income *cohort1916 | 0.0753 | | | 0.0865 | 0.3843 |  | family income *cohort1916 | -0.0604 | 0.0435 | | 0.1646 | |
| education* cohort1926 | -0.3741 | | 0.1515 | | 0.0136 |  | perceived income *cohort1926 | 0.0981 | | | 0.0788 | 0.2133 |  | family income *cohort1926 | -0.0364 | 0.0384 | | 0.3424 | |
| education* cohort1936 | -0.3317 | | 0.1553 | | 0.0328 |  | perceived income *cohort1936 | 0.1270 | | | 0.0787 | 0.1066 |  | family income *cohort1936 | -0.0286 | 0.0377 | | 0.4485 | |
| age | 0.0188 | | 0.0086 | | 0.0281 |  | age | 0.0190 | | | 0.0086 | 0.0268 |  | age | 0.0150 | 0.0091 | | 0.1011 | |
| age square | 0.0007 | | 0.0002 | | 0.0008 |  | age square | 0.0007 | | | 0.0002 | 0.0009 |  | age square | 0.0008 | 0.0002 | | 0.0004 | |
| control variables | yes | | | | |  | control variables | yes | | | | |  | control variables | yes | | | | |
| **random effect** | | | | | |  | **random effect** |  | | | | |  | **random effect** |  | | | | |
| level-1: within-person | 3.5505 | | 0.0490 | | <.0001 |  | level-1: within-person | | 3.5491 | 0.0490 | | <.0001 |  | level-1: within-person | 3.5525 | 0.0490 | <.0001 | | |
| level-2: between-person | | 0.5711 | 0.0386 | <.0001 | |  | level-2:between-person | 0.5748 | | | 0.0387 | <.0001 |  | level-2: between-person | 0.5705 | 0.0387 | | <.0001 | |
| **model fit** | | | | | |  | **model fit** |  | | |  |  |  | **model fit** |  |  | |  | |
| -2LL | 67146 | | | | |  | -2LL | | 67154 | | | |  | -2LL | 67159 | | | | |
| BIC | 67163 | | | | |  | BIC | | 67171 | | | |  | BIC | 67176 | | | | |

| Table S4 Associations between SES, cohort and older adults’ DDS in rural China | | | | | | | | | | | | | | | |
| --- | --- | --- | --- | --- | --- | --- | --- | --- | --- | --- | --- | --- | --- | --- | --- |
|  | Model s1 | | |  |  | Model s2 | | | | |  |  | Model s3 | | |
|  | *β* | SE | *p* |  |  | *β* | | SE | *p* | |  |  | *β* | SE | *p* |
| **fixed effect** |  |  |  |  | **fixed effect** | | |  |  | |  | **fixed effect** | |  |  |
| intercept | 0.2326 | 0.1403 | 0.0973 |  | intercept | | 0.5278 | 0.1811 | 0.0036 | |  | intercept | 0.4493 | 0.2125 | 0.0345 |
| education (ref=illiteracy) | | | |  | education (ref=illiteracy) | | | | | |  | education (ref=illiteracy) | | | |
|  | 0.1985 | 0.0854 | 0.0201 |  |  | | 0.0934 | 0.0351 | 0.0079 | |  |  | 0.0940 | 0.0352 | 0.0075 |
| perceived income | 0.4066 | 0.0217 | <.0001 |  | perceived income | | 0.3281 | 0.0487 | <.0001 | |  | perceived income | 0.4065 | 0.0217 | <.0001 |
| family income | 0.2419 | 0.0099 | <.0001 |  | family income | | 0.2422 | 0.0099 | <.0001 | |  | family income | 0.2246 | 0.0218 | <.0001 |
| cohort (ref=cohort1946) | | | |  | cohort (ref=cohort1946) | | | | | |  | cohort (ref=cohort1946) | | | |
| cohort1916 | -1.9688 | 0.1133 | <.0001 |  | cohort1916 | -2.5539 | | 0.2219 | | <.0001 |  | cohort1916 | -2.4838 | 0.2846 | <.0001 |
| cohort1926 | -1.2659 | 0.0978 | <.0001 |  | cohort1926 | | -1.7944 | 0.1905 | <.0001 | |  | cohort1926 | -1.5164 | 0.2535 | <.0001 |
| cohort1936 | -0.6242 | 0.0893 | <.0001 |  | cohort1936 | | -0.7213 | 0.1848 | <.0001 | |  | cohort1936 | -0.8076 | 0.2372 | 0.0007 |
| education* cohort1916 | -0.2429 | 0.1154 | 0.0354 |  | perceived income *cohort1916 | | 0.1558 | 0.0682 | 0.0224 | |  | family income *cohort1916 | 0.0450 | 0.0309 | 0.1458 |
| education* cohort1926 | -0.1586 | 0.1010 | 0.1162 |  | perceived income *cohort1926 | | 0.1408 | 0.0597 | 0.0183 | |  | family income *cohort1926 | 0.0148 | 0.0270 | 0.5838 |
| education* cohort1936 | -0.0354 | 0.1004 | 0.7243 |  | perceived income *cohort1936 | | 0.0167 | 0.0595 | 0.7796 | |  | family income *cohort1936 | 0.0145 | 0.0259 | 0.5756 |
| age | 0.0554 | 0.0066 | <.0001 |  | age | | 0.0565 | 0.0066 | <.0001 | |  | age | 0.0585 | 0.0069 | <.0001 |
| age square | 0.0002 | 0.0002 | 0.3500 |  | age square | | 0.0001 | 0.0002 | 0.4332 | |  | age square | 0.0001 | 0.0002 | 0.6863 |
| control variables | yes | | |  | control variables | | yes | | | |  | control variables | yes | | |
| **random effect** | | | |  | **random effect** | | | | | |  | **random effect** | |  |  |
| level-1: within-person | 3.5780 | 0.0392 | <.0001 |  | level-1: within-person | | 3.5759 | 0.0392 | <.0001 | |  | level-1: within-person | 3.5775 | 0.0392 | <.0001 |
| level-2: between-person | 0.6450 | 0.0322 | <.0001 |  | level-2: between-person | | 0.6470 | 0.0322 | <.0001 | |  | level-2: between-person | 0.6467 | 0.0323 | <.0001 |
| **model fit** |  |  |  |  | **model fit** | |  |  |  | |  | **model fit** |  | | |
| -2LL | 106028 | | |  | -2LL | | 106027 | | | |  | -2LL | 106041 | | |
| BIC | 106046 | | |  | BIC | | 106045 | | | |  | BIC | 106059 | | |

| \| Table S5 Associations between SES, cohort and older adults’ DDS in urban China \| \| \| \| \| \| \| \| \| \| \| \| \| \| \| \| --- \| --- \| --- \| --- \| --- \| --- \| --- \| --- \| --- \| --- \| --- \| --- \| --- \| --- \| --- \| \|  \| Model s1 \| \| \|  \| \| Model s2 \| \| \| \|  \| Model s3 \| \| \| \| *β* \| SE \| *p* \| *β* \| SE \| *p* \| \| *β* \| SE \| *p* \| \| \| \| **fixed effect** \|  \|  \|  \| \| **fixed effect** \|  \|  \|  \| **fixed effect** \| \|  \|  \|  \| \| \| \| intercept \| 0.1588 \| 0.2091 \| 0.4476 \| \| intercept \| 0.1333 \| 0.2807 \| 0.6348 \| intercept \| \| -0.8862 \| 0.3657 \| 0.0154 \| \| \| \| education (ref=illiteracy) \| \| \|  \| \| education (ref=illiteracy) \| \| \|  \| education (ref=illiteracy) \| \| \| \| \| \| \| \|  \| 0.8654 \| 0.1615 \| <.0001 \| \|  \| 0.1725 \| 0.0453 \| 0.0001 \|  \| \| 0.1733 \| 0.0453 \| 0.0001 \| \| \| \| perceived income \| 0.2675 \| 0.0268 \| <.0001 \| \| perceived income \| 0.4639 \| 0.0794 \| <.0001 \| perceived income \| \| 0.2745 \| 0.0268 \| <.0001 \| \| \| \| family income \| 0.2871 \| 0.0138 \| <.0001 \| \| family income \| 0.2857 \| 0.0138 \| <.0001 \| family income \| \| 0.4619 \| 0.0385 \| <.0001 \| \| \| \| education*age \| -0.0622 \| 0.0144 \| <.0001 \| \| perceived income *age \| -0.0426 \| 0.0065 \| <.0001 \| family income*age \| \| -0.0180 \| 0.0025 \| <.0001 \| \| \| \| cohort (ref=cohort1946) \| \|  \|  \| \| cohort (ref=cohort1946) \| \|  \|  \| cohort (ref=cohort1946) \| \| \|  \|  \| \| \| \| cohort1916 \| -1.6761 \| 0.2114 \| <.0001 \| \| cohort1916 \| -3.3501 \| 0.4671 \| <.0001 \| cohort1916 \| \| -2.4899 \| 0.7192 \| 0.0005 \| \| \| \| cohort1926 \| -1.2323 \| 0.1860 \| <.0001 \| \| cohort1926 \| -2.7022 \| 0.3583 \| <.0001 \| cohort1926 \| \| -1.9120 \| 0.5327 \| 0.0003 \| \| \| \| cohort1936 \| -0.4741 \| 0.1567 \| 0.0025 \| \| cohort1936 \| -1.5250 \| 0.2717 \| <.0001 \| cohort1936 \| \| -0.7650 \| 0.4177 \| 0.0671 \| \| \| \| education* cohort1916 \| -1.0332 \| 0.5428 \| 0.0570 \| \| perceived income *cohort1916 \| -0.0452 \| 0.2224 \| 0.8390 \| family income* cohort1916 \| \| -0.2198 \| 0.1159 \| 0.0579 \| \| \| \| education* cohort1926 \| -0.3841 \| 0.2745 \| 0.1617 \| \| perceived income *cohort1926 \| 0.1065 \| 0.1325 \| 0.4218 \| family income* cohort1926 \| \| -0.1004 \| 0.0713 \| 0.1589 \| \| \| \| education* cohort1936 \| -0.2503 \| 0.1876 \| 0.1822 \| \| perceived income *cohort1936 \| 0.2224 \| 0.0895 \| 0.0130 \| family income* cohort1936 \| \| -0.0157 \| 0.0486 \| 0.7471 \| \| \| \| education* cohort1916*age \| 0.0702 \| 0.0233 \| 0.0026 \| \| perceived income *age*cohort1916 \| 0.0370 \| 0.0083 \| <.0001 \| family income* cohort1916*age \| \| 0.0183 \| 0.0032 \| <.0001 \| \| \| \| education* cohort1926*age \| 0.0467 \| 0.0163 \| 0.0042 \| \| perceived income* age*cohort1926 \| 0.0293 \| 0.0058 \| <.0001 \| family income* cohort1926*age \| \| 0.0137 \| 0.0022 \| <.0001 \| \| \| \| education* cohort1936*age \| 0.0274 \| 0.0121 \| 0.0241 \| \| perceived income* age*cohort1936 \| 0.0128 \| 0.0038 \| 0.0006 \| family income* cohort1936*age \| \| 0.0060 \| 0.0014 \| <.0001 \| \| \| \| age \| 0.0734 \| 0.0151 \| <.0001 \| \| age \| 0.1675 \| 0.0239 \| <.0001 \| age \| \| 0.2042 \| 0.0258 \| <.0001 \| \| \| \| age square \| -0.0002 \| 0.0003 \| 0.4908 \| \| age square \| -0.0014 \| 0.0005 \| 0.0044 \| age square \| \| -0.0022 \| 0.0006 \| 0.0002 \| \| \| \| **random effect** \|  \|  \|  \| \| **random effect** \|  \|  \|  \| **random effect** \| \|  \|  \|  \| \| \| \| level-1: within-person \| 3.5450 \| 0.0489 \| <.0001 \| \| level-1: within-person \| 3.5307 \| 0.0488 \| <.0001 \| level-1: within-person \| \| 3.5275 \| 0.0487 \| <.0001 \| \| \| \| level-2: between-person \| 0.5729 \| 0.0386 \| <.0001 \| \| level-2: between-person \| 0.5850 \| 0.0389 \| <.0001 \| level-2: between-person \| \| 0.5804 \| 0.0388 \| <.0001 \| \| \| \| **model fit** \|  \|  \|  \| \| **model fit** \|  \|  \|  \| **model fit** \| \|  \|  \|  \| \| \| \| -2LL \| 67153 \| \| \| \| -2LL \| 67143 \| \| \| -2LL \| \| 67129 \| \| \| \| \| \| BIC \| 67170 \| \| \| \| BIC \| 67161 \| \| \| BIC \| \| 67146 \| \| \| \| \| |
| --- | --- | --- | --- | --- | --- | --- | --- | --- | --- | --- | --- | --- | --- | --- | --- | --- | --- | --- | --- | --- | --- | --- | --- | --- | --- | --- | --- | --- | --- | --- | --- | --- | --- | --- | --- | --- | --- | --- | --- | --- | --- | --- | --- | --- | --- | --- | --- | --- | --- | --- | --- | --- | --- | --- | --- | --- | --- | --- | --- | --- | --- | --- | --- | --- | --- | --- | --- | --- | --- | --- | --- | --- | --- | --- | --- | --- | --- | --- | --- | --- | --- | --- | --- | --- | --- | --- | --- | --- | --- | --- | --- | --- | --- | --- | --- | --- | --- | --- | --- | --- | --- | --- | --- | --- | --- | --- | --- | --- | --- | --- | --- | --- | --- | --- | --- | --- | --- | --- | --- | --- | --- | --- | --- | --- | --- | --- | --- | --- | --- | --- | --- | --- | --- | --- | --- | --- | --- | --- | --- | --- | --- | --- | --- | --- | --- | --- | --- | --- | --- | --- | --- | --- | --- | --- | --- | --- | --- | --- | --- | --- | --- | --- | --- | --- | --- | --- | --- | --- | --- | --- | --- | --- | --- | --- | --- | --- | --- | --- | --- | --- | --- | --- | --- | --- | --- | --- | --- | --- | --- | --- | --- | --- | --- | --- | --- | --- | --- | --- | --- | --- | --- | --- | --- | --- | --- | --- | --- | --- | --- | --- | --- | --- | --- | --- | --- | --- | --- | --- | --- | --- | --- | --- | --- | --- | --- | --- | --- | --- | --- | --- | --- | --- | --- | --- | --- | --- | --- | --- | --- | --- | --- | --- | --- | --- | --- | --- | --- | --- | --- | --- | --- | --- | --- | --- | --- | --- | --- | --- | --- | --- | --- | --- | --- | --- | --- | --- | --- | --- | --- | --- | --- | --- | --- | --- | --- | --- | --- | --- | --- | --- | --- | --- | --- | --- | --- | --- | --- | --- | --- | --- | --- | --- | --- | --- | --- | --- | --- | --- | --- | --- | --- | --- | --- | --- | --- | --- | --- | --- | --- | --- | --- | --- | --- | --- | --- | --- | --- | --- | --- | --- | --- | --- | --- | --- | --- | --- | --- | --- | --- | --- | --- | --- | --- | --- | --- | --- | --- | --- | --- | --- | --- | --- | --- | --- | --- | --- | --- | --- | --- | --- | --- | --- | --- | --- | --- | --- | --- | --- | --- | --- | --- | --- | --- | --- | --- | --- | --- | --- | --- | --- | --- | --- | --- | --- | --- | --- | --- | --- | --- | --- | --- | --- | --- | --- | --- | --- | --- | --- | --- | --- | --- | --- | --- | --- | --- | --- | --- | --- | --- | --- | --- | --- | --- | --- | --- | --- | --- | --- | --- | --- | --- | --- | --- | --- | --- | --- | --- | --- | --- | --- | --- | --- | --- | --- | --- | --- | --- | --- | --- | --- | --- | --- | --- | --- | --- | --- | --- | --- | --- | --- | --- |

| Table S6 Associations between SES, cohort and older adults’ DDS in rural China | | | | | | | | | | | | | |
| --- | --- | --- | --- | --- | --- | --- | --- | --- | --- | --- | --- | --- | --- |
|  | Model s1 | | |  | Model s2 | | |  | Model s3 | | |  |  |
|  | *β* | SE | *p* |  | *β* | SE | *p* |  | *β* | SE | *p* |  |  |
| **fixed effect** |  |  |  | **fixed effect** |  |  |  | **fixed effect** |  |  |  |  |  |
| intercept | -0.0328 | 0.1449 | 0.8210 | intercept | -0.3051 | 0.2079 | 0.1422 | intercept | -0.8962 | 0.2455 | 0.0003 |  |  |
| education (ref=illiteracy) | |  |  | education (ref=illiteracy) | |  |  | education (ref=illiteracy) | |  |  |  |  |
|  | 0.6503 | 0.1073 | <.0001 |  | 0.1090 | 0.0352 | 0.0020 |  | 0.1116 | 0.0352 | 0.0015 |  |  |
| perceived income | 0.4124 | 0.0217 | <.0001 | perceived income | 0.6416 | 0.0606 | <.0001 | perceived income | 0.4223 | 0.0217 | <.0001 |  |  |
| family income | 0.2356 | 0.0099 | <.0001 | family income | 0.2314 | 0.0099 | <.0001 | family income | 0.3749 | 0.0266 | <.0001 |  |  |
| cohort (ref=cohort1946) | |  |  | cohort (ref=cohort1946) | |  |  | cohort (ref=cohort1946) | |  |  |  |  |
| cohort1916 | -2.4542 | 0.1383 | <.0001 | cohort1916 | -3.2925 | 0.3612 | <.0001 | cohort1916 | -3.2393 | 0.4909 | <.0001 |  |  |
| cohort1926 | -1.7157 | 0.1167 | <.0001 | cohort1926 | -2.6712 | 0.2678 | <.0001 | cohort1926 | -2.4179 | 0.3559 | <.0001 |  |  |
| cohort1936 | -0.8550 | 0.0946 | <.0001 | cohort1936 | -1.2043 | 0.2000 | <.0001 | cohort1936 | -1.1833 | 0.2689 | <.0001 |  |  |
| education* cohort1916 | -1.1680 | 0.4995 | 0.0194 | perceived income *cohort1916 | -0.7867 | 0.1861 | <.0001 | family income* cohort1916 | -0.3830 | 0.0898 | <.0001 |  |  |
| education* cohort1926 | -0.2884 | 0.2242 | 0.1983 | perceived income *cohort1926 | -0.1350 | 0.1060 | 0.2030 | family income* cohort1926 | -0.1273 | 0.0535 | 0.0174 |  |  |
| education* cohort1936 | 0.0131 | 0.1348 | 0.9229 | perceived income *cohort1936 | -0.0177 | 0.0687 | 0.7974 | family income* cohort1936 | -0.0141 | 0.0344 | 0.6817 |  |  |
| education*age | -0.0696 | 0.0099 | <.0001 | perceived income* age | -0.0426 | 0.0050 | <.0001 | family income*age | -0.0186 | 0.0019 | <.0001 |  |  |
| education* cohort1916*age | 0.0853 | 0.0192 | <.0001 | perceived income *age*cohort1916 | 0.0633 | 0.0066 | <.0001 | family income* cohort1916*age | 0.0267 | 0.0026 | <.0001 |  |  |
| education* cohort1926*age | 0.0556 | 0.0125 | <.0001 | perceived income *age*cohort1926 | 0.0414 | 0.0046 | <.0001 | family income* cohort1926*age | 0.0174 | 0.0018 | <.0001 |  |  |
| education* cohort1936*age | 0.0310 | 0.0093 | 0.0008 | perceived income *age*cohort1936 | 0.0220 | 0.0029 | <.0001 | family income* cohort1936*age | 0.0085 | 0.0011 | <.0001 |  |  |
| age | 0.1117 | 0.0101 | <.0001 | age | 0.2102 | 0.0181 | <.0001 | age | 0.2555 | 0.0182 | <.0001 |  |  |
| age square | -0.0008 | 0.0002 | 0.0003 | age square | -0.0031 | 0.0004 | <.0001 | age square | -0.0039 | 0.0004 | <.0001 |  |  |
| **random effect** |  |  |  | **random effect** |  |  |  | **random effect** |  |  |  |  |  |
| level-1: within-person | 3.5664 | 0.0391 | <.0001 | level-1: within-person | 3.5534 | 0.0390 | <.0001 | level-1: within-person | 3.5471 | 0.0390 | <.0001 |  |  |
| level-2: between-person | 0.6495 | 0.0323 | <.0001 | level-2: between-person | 0.6550 | 0.0323 | <.0001 | level-2: between-person | 0.6508 | 0.0322 | <.0001 |  |  |
| **model fit** |  |  |  | **model fit** |  |  |  | **model fit** |  |  |  |  |  |
| -2LL | 106002 | | | -2LL | 105957 | | | -2LL | 105912 | | |  |  |
| BIC | 106020 | | | BIC | 105975 | | | BIC | 105930 | | |  |  |

#
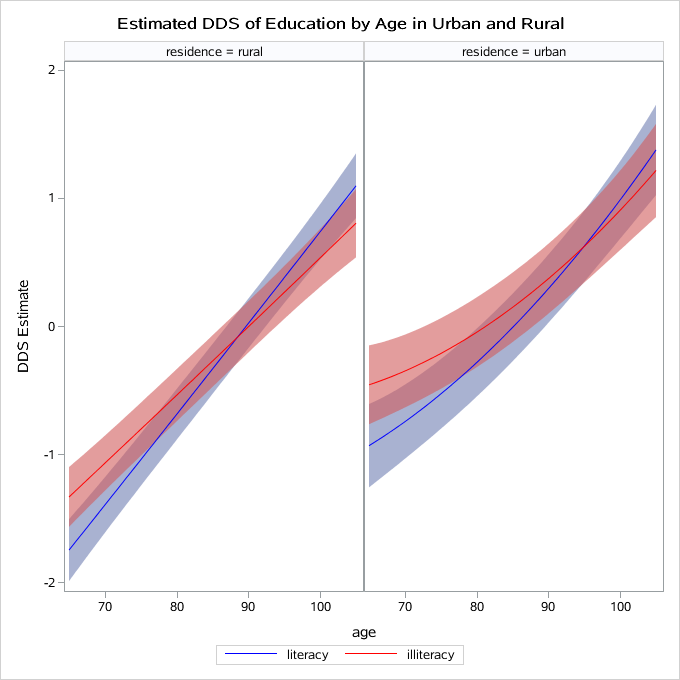

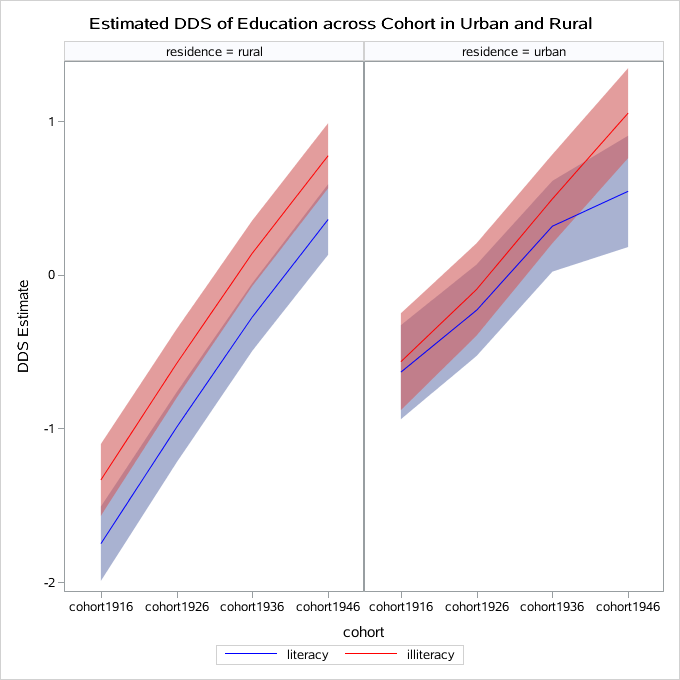
Figure S1 Estimated DDS of education with age and across successive cohorts in urban and rural older adults

#
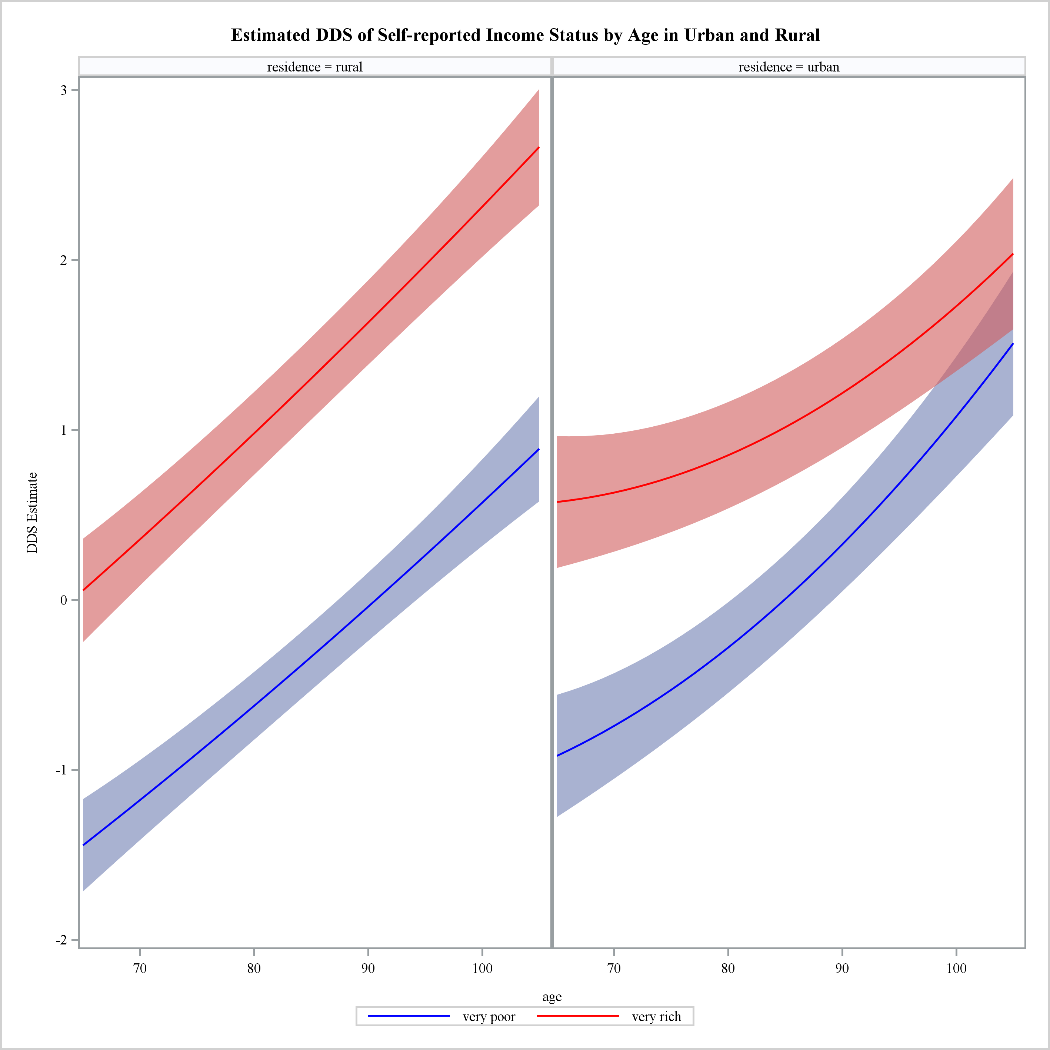

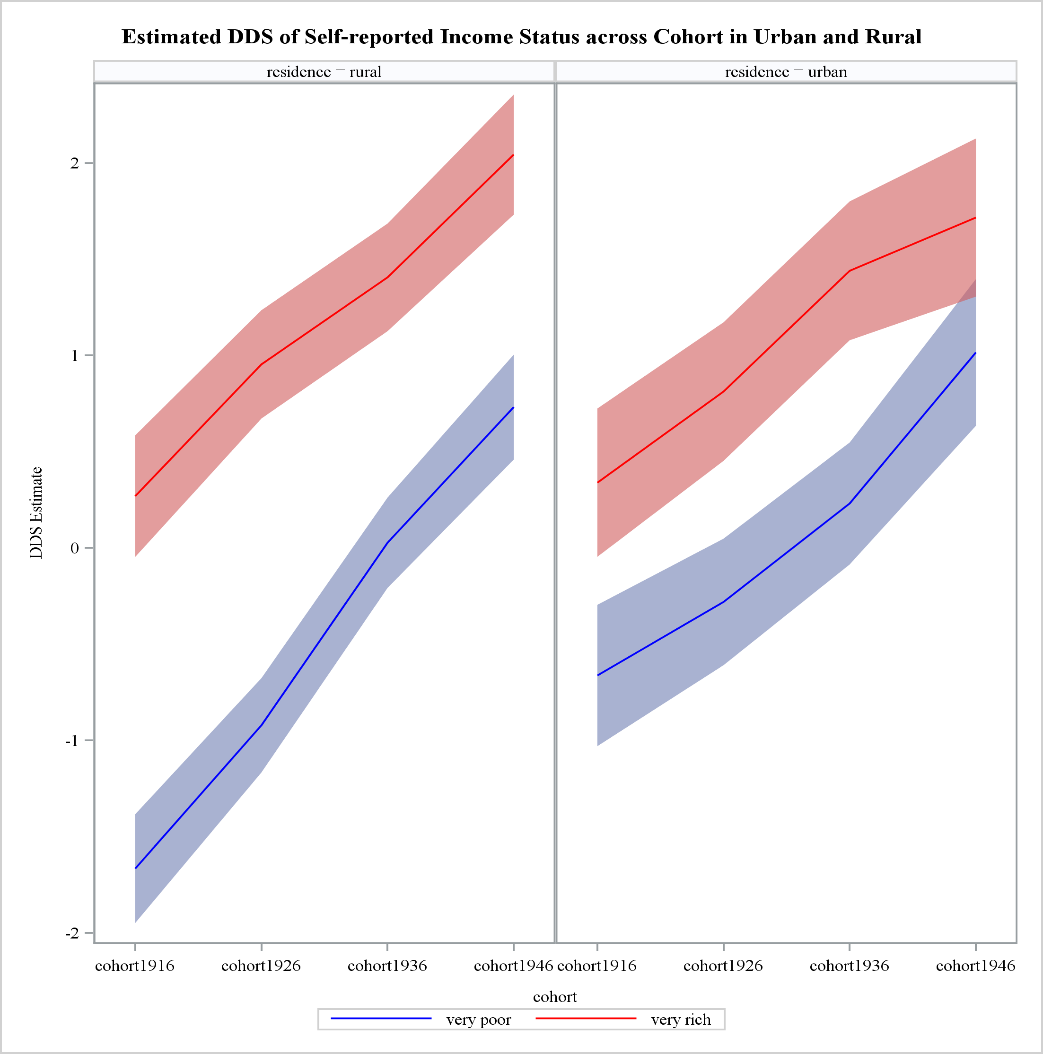
Figure S2 Estimated DDS of perceived or self-reported income status with age and across successive cohorts in urban and rural older adults

#
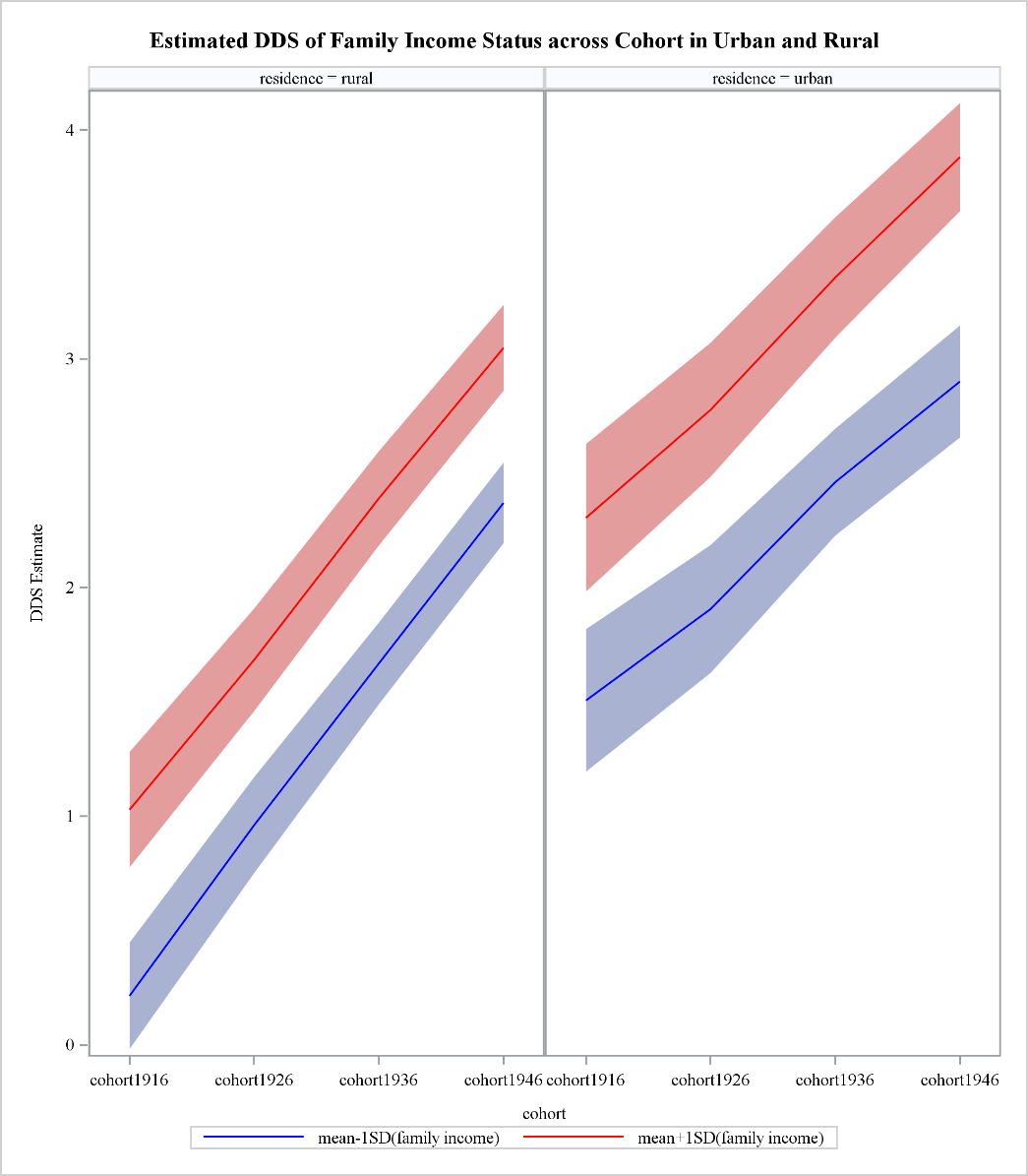

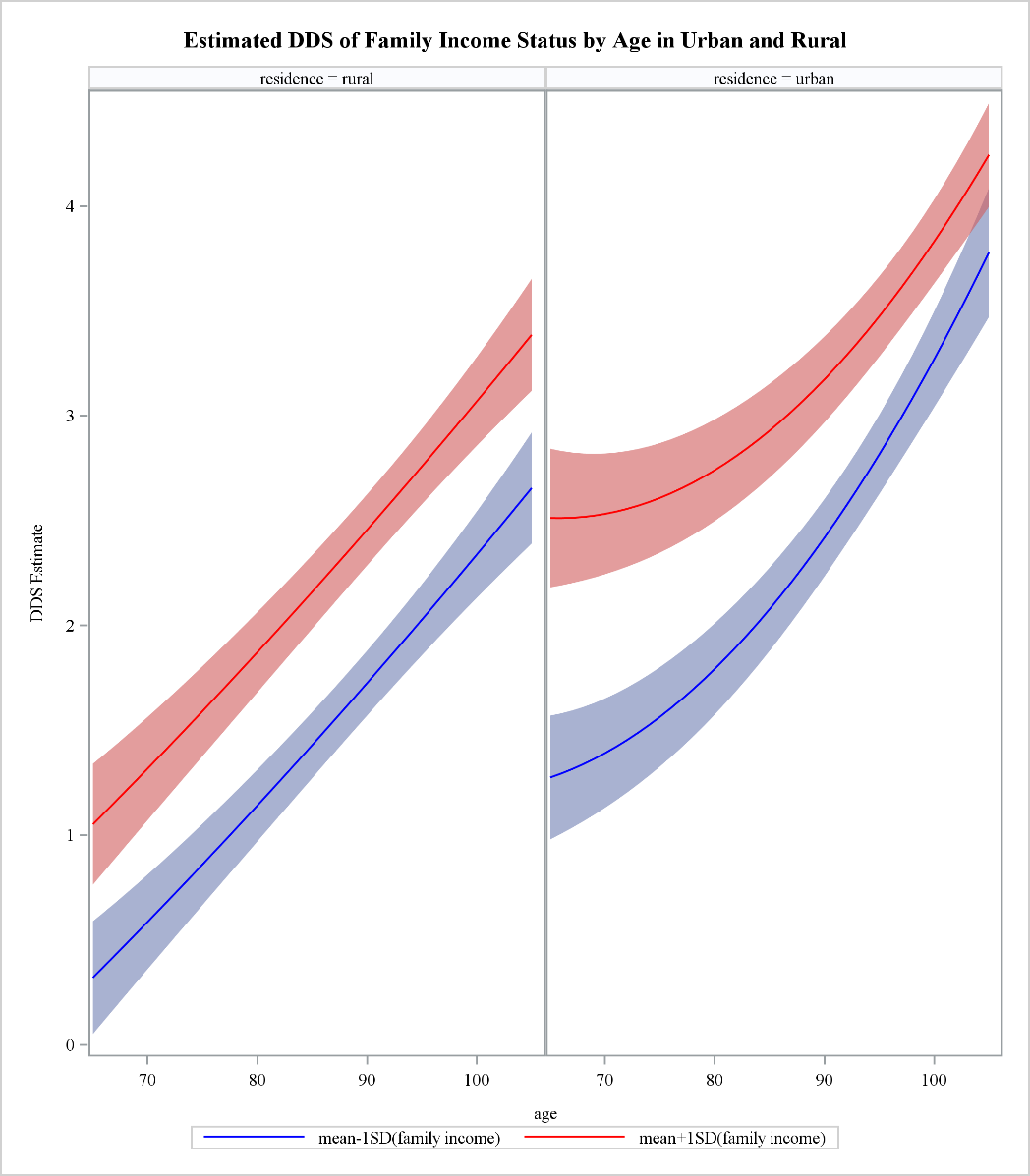
Figure S3 Estimated DDS of family income with age and across successive cohorts in urban and rural older adults

**
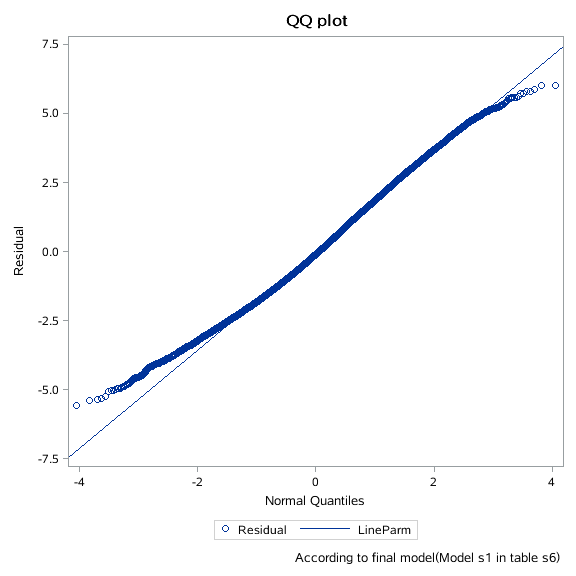

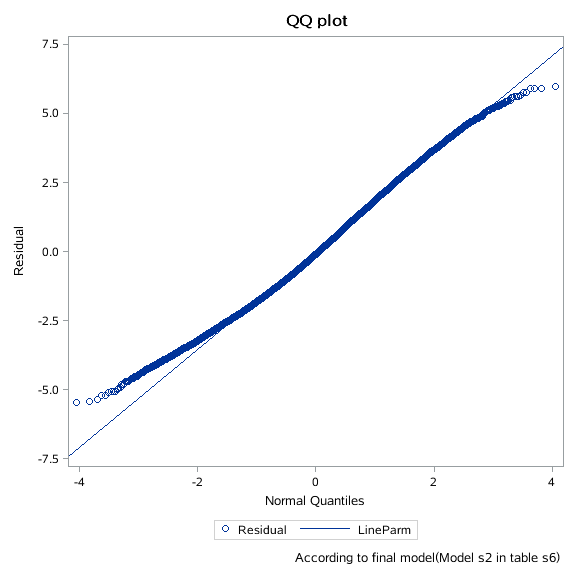

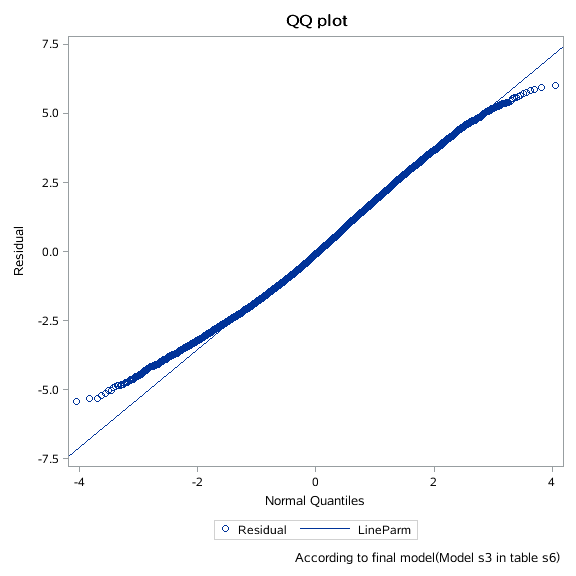

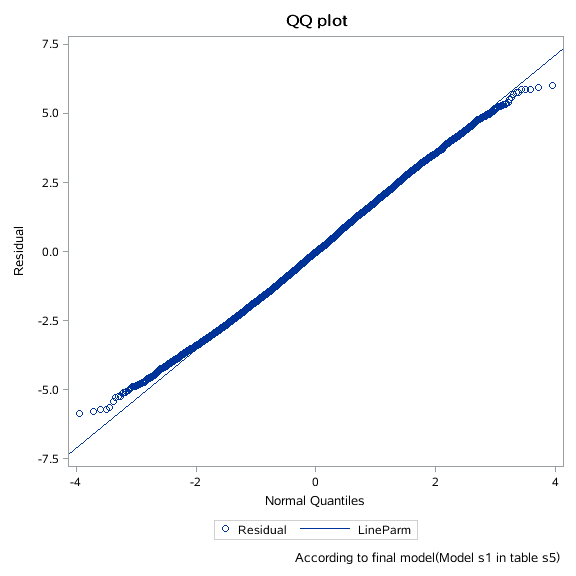

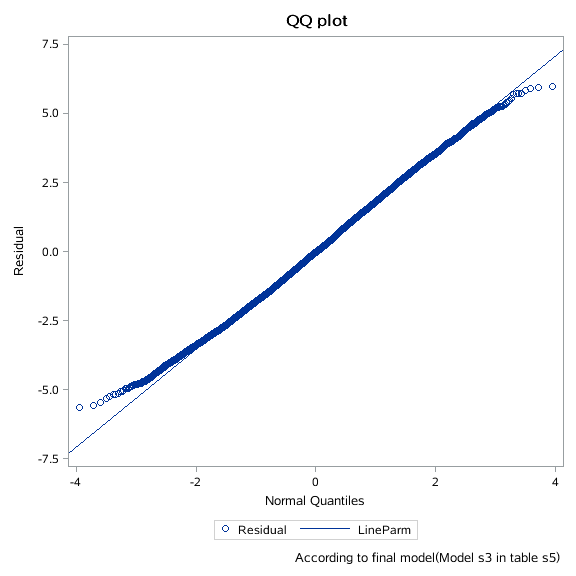

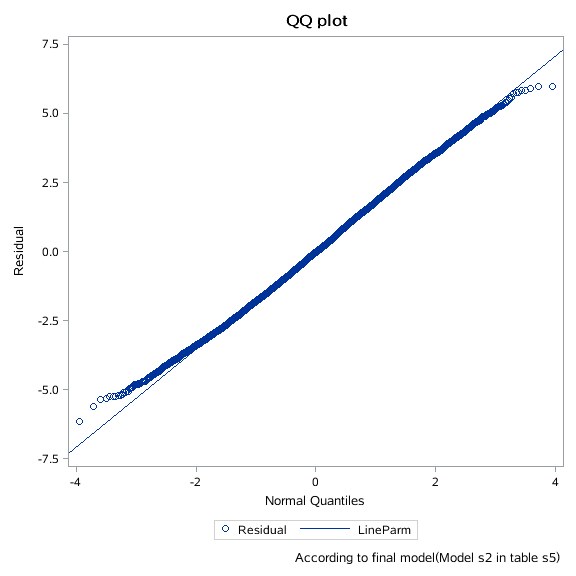
Figure S4 Normality test for models’ residuals using Q-Q plots**
